# Dynamic Immune Cell Recruitment After Murine Pulmonary *Aspergillus fumigatus* Infection under Different Immunosuppressive Regimens

**DOI:** 10.3389/fmicb.2016.01107

**Published:** 2016-07-13

**Authors:** Natarajaswamy Kalleda, Jorge Amich, Berkan Arslan, Spoorthi Poreddy, Katharina Mattenheimer, Zeinab Mokhtari, Hermann Einsele, Matthias Brock, Katrin Gertrud Heinze, Andreas Beilhack

**Affiliations:** ^1^Department of Medicine II, Würzburg University HospitalWürzburg, Germany; ^2^Research Center for Infectious Diseases, Julius-Maximilians-University WürzburgWürzburg, Germany; ^3^Interdisciplinary Center for Clinical Science Research LaboratoryWuürzburg, Germany; ^4^Graduate School of Life Sciences WürzburgWürzburg, Germany; ^5^Max Planck Institute for Chemical EcologyJena, Germany; ^6^Leibniz Institute for Natural Product Research and Infection Biology, Hans Knoell Institute, Friedrich Schiller University JenaJena, Germany; ^7^Institute for Microbiology, Friedrich Schiller University JenaJena, Germany; ^8^Fungal Genetics and Biology Group, University of Nottingham, School of Life SciencesNottingham, UK; ^9^Rudolf Virchow Center, Julius-Maximilians-University WürzburgWürzburg, Germany

**Keywords:** *Aspergillus fumigatus*, immune cell recruitment, CD11b^+^ myeloid cells, corticosteroids and cyclophosphamide

## Abstract

Humans are continuously exposed to airborne spores of the saprophytic fungus *Aspergillus fumigatus*. However, in healthy individuals pulmonary host defense mechanisms efficiently eliminate the fungus. In contrast, *A. fumigatus* causes devastating infections in immunocompromised patients. Host immune responses against *A. fumigatus* lung infections in immunocompromised conditions have remained largely elusive. Given the dynamic changes in immune cell subsets within tissues upon immunosuppressive therapy, we dissected the spatiotemporal pulmonary immune response after *A. fumigatus* infection to reveal basic immunological events that fail to effectively control invasive fungal disease. In different immunocompromised murine models, myeloid, notably neutrophils, and macrophages, but not lymphoid cells were strongly recruited to the lungs upon infection. Other myeloid cells, particularly dendritic cells and monocytes, were only recruited to lungs of corticosteroid treated mice, which developed a strong pulmonary inflammation after infection. Lymphoid cells, particularly CD4^+^ or CD8^+^ T-cells and NK cells were highly reduced upon immunosuppression and not recruited after *A. fumigatus* infection. Moreover, adoptive CD11b^+^ myeloid cell transfer rescued cyclophosphamide immunosuppressed mice from lethal *A. fumigatus* infection but not cortisone and cyclophosphamide immunosuppressed mice. Our findings illustrate that CD11b^+^ myeloid cells are critical for anti-*A. fumigatus* defense under cyclophosphamide immunosuppressed conditions.

## Introduction

Respiratory lung infections caused by the pathogenic mold *Aspergillus fumigatus* result in life-threatening complications in immunocompromised patients, for instance, after allogeneic hematopoietic cell transplantation, solid organ transplantation, chemotherapy for cancer, or other acquired or congenital immune disorders (Nihtinen et al., 2010; Kousha et al., 2011; Singh et al., 2013). *A. fumigatus* is a ubiquitous, airborne saprophytic fungus, which produces thousands of conidia with every conidiophore (Latge, 1999). The conidia are rapidly released into the environment. Their hydrophobic exterior and small diameter of 2–3 μm facilitates them to reach the lung alveoli easily by crossing physiological barriers (Latge, 1999; Dagenais and Keller, 2009; Park and Mehrad, 2009). However, healthy individuals do not develop invasive lung infections despite a continuous exposure to fungal spores (Garcia-Vidal et al., 2013) without signs of antibody- or cell-mediated adaptive immune response or symptoms attributable to *A. fumigatus* inhalation (Park and Mehrad, 2009). A steadily increasing population of immunocompromised patients is at greater risk and experiences life-threatening invasive infections by *A. fumigatus*. Although, several antifungal drugs have become available to combat *A. fumigatus* infections, the mortality of this devastating disease remains as high as 90% in immunocompromised patients (Dagenais and Keller, 2009). Efforts to improve management and treatment of *A. fumigatus* lung infections are mostly concentrated on identification of new antifungal drug targets and compounds (Segal et al., 2006). However, it appears essential to develop therapies that improve the host immune defense in immunocompromised patients. To this end, an in-depth understanding of the dynamic host immune responses against *A. fumigatus* lung infections under immunocompromised condition is a prerequisite for successful applications of novel therapeutic strategies to effectively manage and treat lung infections in high-risk immunocompromised patients.

Due to various clinical therapies, patient numbers requiring the administration of immunosuppressive drugs are constantly increasing. The most commonly used immunosuppressive drugs in clinical situations with various conditions are cyclophosphamide and corticosteroids (Barnes, 2006; Emadi et al., 2009; Shaikh et al., 2012). Cyclophosphamide is a widely used antineoplastic drug and potent immunosuppressive agent used in the treatment for a wide range of diseases such as solid tumors, hematologic malignancies, autoimmune disorders and as a conditioning regimen for stem cell mobilization and hematopoietic cell transplantation (Emadi et al., 2009). Corticosteroids have proven as most effective anti-inflammatory treatment for asthma and for a number of other inflammatory and immune diseases (Barnes, 2006). Some clinical therapies also use a combination of cyclophosphamide and corticosteroids (Thone et al., 2008). The differences in *A. fumigatus* infection and inflammatory response in corticosteroid and chemotherapeutic models of invasive aspergillosis have been addressed previously; however these analyses focused on immune cells and cytokines contained in the bronchoalveolar lavage fluid after infection (Balloy et al., 2005). Despite the widespread clinical use, knowledge remains limited on how these immunosuppressive treatments modulate immune cell recruitment after lethal *A. fumigatus* lung infection.

The most frequent source of invasive pulmonary infection is the inhalation of conidia into the lungs and sinuses (Latge, 1999). The small size of *A. fumigatus* conidia and their hydrophobic protein coat layer conceals immune stimulatory polysaccharides and protect them from host defense (Enoch et al., 2006; Aimanianda et al., 2009). The virulence of *A. fumigatus* is multifactorial, and it depends on both host and fungal properties (Abad et al., 2010). However, host immune status is a key determinant for the initiation and outcome of infection. Host immunosuppression allows the germination of *A. fumigatus* conidia and subsequent development to hyphae, which leads to invasive lung infection. Therefore, the host immune response at the site of infection is a key factor for the fate of *A. fumigatus* conidia in the lung tissue. However, the timing and magnitude of host immune cell responses following *A. fumigatus* conidial inhalation, as well as continuous host defense throughout the different developmental stages of fungi in immunocompromised conditions remain poorly defined. The innate immune response is crucial to clear *A. fumigatus* infection (Margalit and Kavanagh, 2015). The adoptive transfer of myeloid progenitors protect against *A. fumigatus* infections in chemically induced neutropenic mouse models (BitMansour et al., 2002; BitMansour et al., 2005) and this protective effect is mediated across major histocompatibility complex barriers (Arber et al., 2005). However, transfused myeloid precursors have to differentiate into effector cells to fight against infection. In contrast, adoptively transferred terminally differentiated myeloid cells may not survive for longer time periods to completely clear infection. Thus, the transfusion of a mixed myeloid population that consists of undifferentiated precursors and terminally differentiated effector cells might be an ideal approach to fight against *A. fumigatus* infections. However, to date this approach has not been investigated. Here, we employed a combination of murine *in vivo* models to investigate immune cell responses following respiratory fungal challenge with *A. fumigatus* conidia under different immunosuppressive regimens. We show that CD11b^+^ myeloid cells are critical for anti-*A. fumigatus* defense in immunocompromised conditions and that adoptive CD11b^+^ myeloid cell transfer into cyclophosphamide immunosuppressed mice rescues mice from lethal *A. fumigatus* infection.

## Materials and Methods

### Animals

Inbred BALB/c female mice were purchased from Charles River (Sulzfeld, Germany) and maintained in the pathogen-free animal facility of the Institute for Molecular Infection Biology (IMIB), University of Würzburg, Germany. Firefly luciferase transgenic BALB/c.L2G85 female mice had been backcrossed from FVB/N.L2G85 mice for more than 12 generations (Cao et al., 2004; Beilhack et al., 2005). All experiments were performed with 8–12-week-old female mice. All animal experiments were carried out according to German guidelines for animal experimentation. The responsible authority (Regierung von Unterfranken; Permit Number 55.2-2531.01-86-13) approved the study.

### Immunosuppressive Treatments

In the cyclophosphamide and corticosteroid treated (CCT) model, mice were intraperitoneally injected with 150 mg kg^-1^ cyclophosphamide (Sigma–Aldrich, Munich, Germany) and subcutaneously (s.c.) with 112 mg kg^-1^ hydrocortisone acetate (Sigma–Aldrich) on days -3 and -1 before *A. fumigatus* infection. In the corticosteroid treated (CT) model, mice were s.c. injected with 112 mg kg^-1^ hydrocortisone acetate on days -3 and -1 before infection.

### Fungal Strains and Infection

The clinical isolate of *A. fumigatus* ATCC46645 strain (Hearn and Mackenzie, 1980) was routinely used. Fluorescent *A. fumigatus* strain Afu-TdTomato (Lother et al., 2014) generated from ATCC46645 (kindly provided by Dr. Sven Krappmann) was used to determine fungal developmental stages inside lung tissue. All the fungal strains were cultivated on defined minimal medium (Amich et al., 2013) under standard culture conditions and handled according to German laboratory safety guidelines. Conidia were harvested from sporulating mycelium using the standard saline/0.01% tween solution, filtered through cell strainer and finally washed with sterile saline. Mice were anesthetized by intraperitoneal injection of ketamine (50 μg/g bodyweight) and xylacine (5 μg/g bodyweight) in 0.1 M phosphate-buffered saline (PBS) in a total volume of 10 μl/g bodyweight and intra-nasally infected with 1 × 10^6^ conidia suspended in 50 μl saline/0.01% tween. All infected mice were monitored carefully according to the standard guidelines; briefly, mice were regularly observed twice a day and carefully monitored for weight loss and disease symptoms. In the immune cell recruitment studies at least *n* = 3/group of mice were used in each independent experiment and data are pooled from three different experiments. In survival studies at least *n* = 5/group of mice were used in each independent experiment.

### Preparation of Lung Single Cell Suspensions for FACS

Single cell suspensions were prepared from lungs according to the previously described protocol (Stockmann et al., 2010) with some modifications. Briefly, left and right lung lobes were dissected from euthanized mice and finely minced using surgical blades in six well-tissue culture plates containing RPMI medium (Life Technologies, USA), then enzymatically digested for 30 min at 37°C in presence of 2 mg/ml Collagenase D and 0.1 mg/ml DNase I (Roche, Mannheim, Germany), diluted with PBS + 0.5 % BSA, filtered through a 70 μm cell strainer (Greiner bio-one, Frickenhausen, Germany) and centrifuged at 1200 rpm for 5 min. The lung cell pellet was re-suspended in erythrocyte lysis buffer (168 mM NH_4_Cl, 10 mM KHCO_3_, 0.1 mM ethylene diamine tetra acetic acid (EDTA)) for 2 min, and immediately diluted with double the volume of PBS and centrifuged. Finally, single cell suspensions were diluted to desired volumes suitable for flow cytometry analyses.

### Flow Cytometry Analysis

Cells were blocked with normal rat serum (1: 20 in PBS) and stained with appropriate antibodies at 4°C for 30 min. To discriminate live/dead cells, they were stained with LIVE/DEAD fixable violet dead cell stain kit (Invitrogen). All the antibodies used were from Biolegend (Uithoorn, The Netherlands). Antibodies (clones) utilized are listed below: CD90.2-PE (30-H12), CD4-APC/Cy7 (GK1.5), CD8-APC/Cy7 (53–6.7), CD11b-perCP-Cy5.5 (M1/70), CD11b-PE (M1/70) CD11c-FITC (N418), I-A/I-E-PE/Cy7 (M5/114.15.2), SiglecF-APC (E50-2440), Ly-6G-APC (1A8), FITC-Ly-6C (HK1.4), Ly6C-PerCP-Cy5.5 (HK1.4), F4/80-APC/Cy7 (BM8), CD49b-PE/Cy7 (DX5). All experiments were carried out using a BD fluorescence-activated cell sorting (FACS) Canto II (BD Biosciences) and data was recorded using BD FACS Diva software and analyzed using FlowJo software version 8.0 (Tree Star, Ashland, OR, USA).

### Immunofluorescence Microscopy

Cryo-embedded lung tissues were cut into 8 μm thick sections on a Leica CM1900 cryostat (Leica Microsystems, Wetzlar, Germany) and mounted onto frosted slides. Slides were air-dried and fixed with acetone at room temperature for 7 min. Slides were counterstained with 4′,6-diamidino-2-phenylindole (DAPI) and mounted with mounting medium (Vector Laboratories, Peterborough, UK) or stained with hematoxylin and eosin. Luciferase expressing CD11b^+^ myeloid cells were stained with an anti-luciferase antibody (Abcam, USA) and the secondary Goat anti-Rabbit IgG, FITC-conjugated antibody (Abcam, USA) according to the manufacturer’s instructions. To detect apoptotic cells TUNEL staining was performed with a commercial kit (Roche Diagnostics, Mannheim, Germany) according to manufacturer’s instructions. Images were taken using Z1 fluorescence microscope (Carl Zeiss, Gottingen, Germany) and evaluated with Zeiss AxioVision software (Carl Zeiss).

### Cytometric Bead Array

Lungs were homogenized in 500 μl PBS using Precellys ceramic kit 1.4 mm in a Precellys 24 homogenizer. Serum was separated from cell debris by 10 min centrifugation at 13000 rpm 4°C and immediately stored at -80°C until further use. Cytokine/chemokine concentrations were measured using BD Cytometric Bead Array Kit (BD Pharmingen, Heidelberg, Germany) or Biolegend Multiplex assay kit (Biolegend, Uithoorn, The Netherlands) according to the manufacturer’s instructions. Data were analyzed by FCAP Array v2.0 software.

### Isolation of CD11b^+^ Myeloid Cells and Adoptive Transfer

Mouse CD11b^+^ myeloid cells were enriched from bone marrow (flushed from femur and tibia bones with PBS) of healthy untreated or hydrocortisone-treated BALB/c mice, using myeloid cell enrichment kit (STEMCELL Technologies, Cologne, Germany) according to manufacturer’s instructions. Cell purity was confirmed by post-enrichment FACS analysis (>90%) in all the experiments. Enriched cells were adoptively transferred via retro-orbital i.v. injection after mice were anesthetized by intraperitoneal injection of ketamine (50 μg/g bodyweight) and xylacine (5 μg/g bodyweight) in 0.1 M Phosphate-Buffered Saline (PBS) in a total volume of 10 μl/g bodyweight.

### Bioluminescence Imaging

*Ex vivo* lung bioluminescence imaging was performed as previously described (Chopra et al., 2013, 2015). Briefly, mice were injected with 300 mg/kg D-luciferin and euthanized after 10 min to prepare lungs and immediately subjected to *ex vivo* bioluminescence imaging with an IVIS Spectrum imaging system (Perkin–Elmer/Caliper Life Sciences, Mainz, Germany). Images were evaluated with Living Image 4.0 software (Caliper Life Sciences).

### Statistical Analyses

All the measurements are expressed as the mean ± standard deviation (SD). Statistical analyses were performed using Graph Pad Prism 6 (Groningen, The Netherlands) software. To compare cell numbers between the two different groups the unpaired Mann–Whitney *u*-test was applied. Significant differences are marked as follows: ^∗^*P* < 0.05; ^∗∗^*P* < 0.01; ^∗∗∗^*P* < 0.001. To compare survival curves of infected mice, the Log-rank (Mantel-Cox) test was utilized.

## Results

### Immunocompromised Murine Models to Study *A. fumigatus* Lung Infections

**FIGURE 1:**
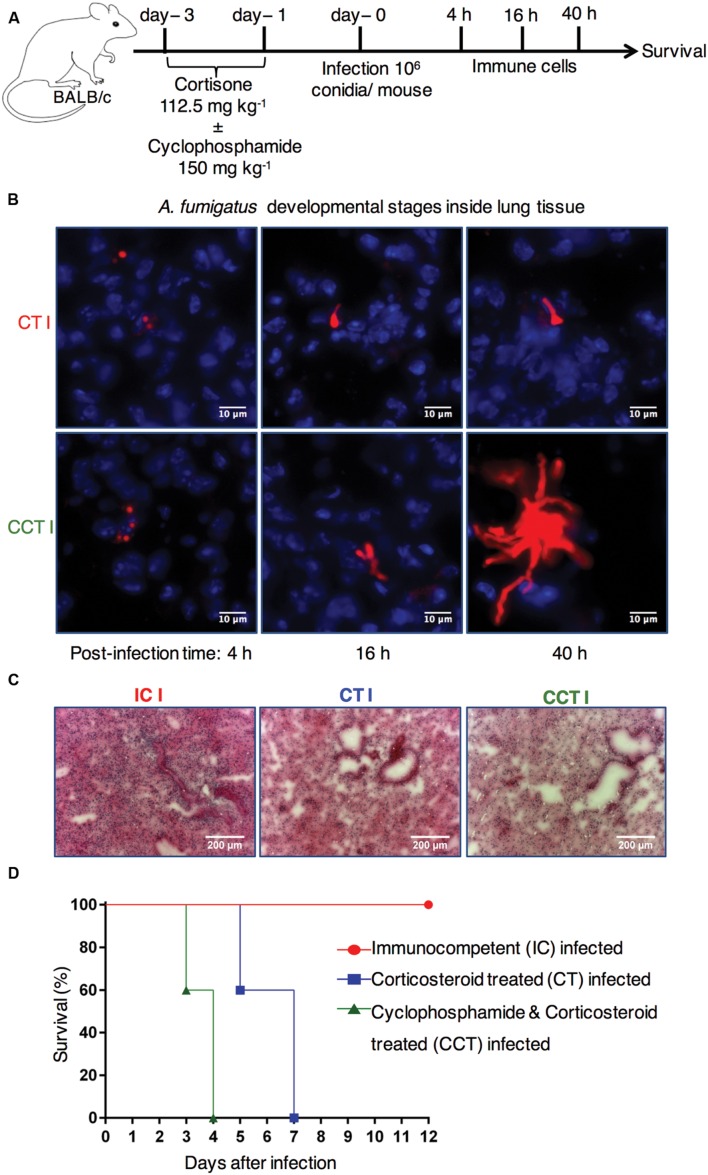
**Immunocompromised mouse models to investigate the dynamic host immune response and survival after *Aspergillus fumigatus* infection.** **(A)** Experimental setup. BALB/c mice were treated with hydrocortisone (112.5 mg kg^-1^) on day -3 and day -1 (CT mice) or with cyclophosphamide (150 mg kg^-1^) and hydrocortisone (112.5 mg kg^-1^) on day -3 and day -1 before *A. fumigatus* infection (CCT mice). On day 0 mice were intranasally infected with 1 × 10^6^ conidia/mouse. Pulmonary immune cell and cytokine responses were analyzed at 4, 16, and 40 h post infection (p.i.). Survival was followed for 12 days p.i. **(B)** *A. fumigatus* developmental stages inside lung tissue. Immunofluorescence microscopy of lungs from immunosuppressed mice that were infected with Afu-TdTomato conidia at 4, 16, and 40 h p.i. Upper panel CT mice and lower panel CCT mice, *A. fumigatus* in red color and 4′,6-diamidino-2-phenylindole (DAPI) staining for nuclei in blue color. Scale bar 10 μM. **(C)** Lung immune cell infiltration in IC, CT, and CCT infected mice: Lung sections were stained with hematoxylin & eosin at 40 h p.i. and imaged in bright field microscope. Scale bar 200 μM. **(D)** Survival of mice under different immunosuppressive regimens: immunocompetent infected (IC infected), corticosteroid treated and infected (CT infected), and cyclophosphamide and corticosteroid treated and infected (CCT infected); (*n* = 5/group). Immunocompetent mice (IC) are resistant to infection, whereas CT (*P* = 0.0004) and CCT (*P* < 0.0001) mice succumb to invasive aspergillosis. However, CT mice survive infection significantly longer than CCT mice (*P* < 0.0001). When mice lost ≥20% weight, they reached an experimental end point and were euthanized according to animal ethics regulations. Log-rank (Mantel–Cox) test was utilized to determine differences in survival.

To explore how immunosuppressive therapy affects pulmonary control of *A. fumigatus* infection, we compared immunocompetent mice with two different immunosuppressed mouse models (**Figure 1A**). Firstly, cyclophosphamide and cortisone treated (CCT) mice and, secondly, corticosteroid treated (CT) mice to investigate pulmonary host immune responses following respiratory *A. fumigatus* infection. We examined different morphotypes of fungal developmental stages in infected lung sections at 4, 16, and 40 h post-infection (p.i.) time points with immunofluorescence microscopy. We observed fungal differentiation from conidia at 4 h p.i. to germlings at 16 h p.i., and hyphae at 40 h p.i. in CT infected lung sections and CCT infected lung sections (**Figure 1B**). However, we observed elongated filaments (hyphal growth) in CCT mice at 16 h and more clearly at 40 h (**Figure 1B**) compared to CT infected mice. Strikingly, these results suggested that different numbers or types of immune cells might have been recruited to lungs of CT infected mice to restrict the hyphal growth. Qualitative hematoxylin & eosin staining of lung sections from immunocompetent, CT and CCT mice at 40 h p.i. exhibited different levels of infiltrated lung immune cells. Lung sections from immunocompetent mice revealed a strong pulmonary immune cell infiltration, CT mice showed less infiltration compared to immunocompetent mice, whereas CCT mice showed fewer infiltrating immune cells (**Figure 1C**). Next, we infected immunocompetent, CCT and CT mice with *A. fumigatus* conidia to determine their survival after *A. fumigatus* infection. Immunocompetent mice were resistant to infection, whereas CCT mice survived until 4 days p.i. and CT mice survived until 7 days p.i. (**Figure 1D**). We hypothesized that some immune cells would have been recruited to the infected lungs to fight against infection in these immunocompromised mouse models.

### Neutrophils and Macrophages are Actively Recruited to Infected Lungs in CCT Mice

**FIGURE 2:**
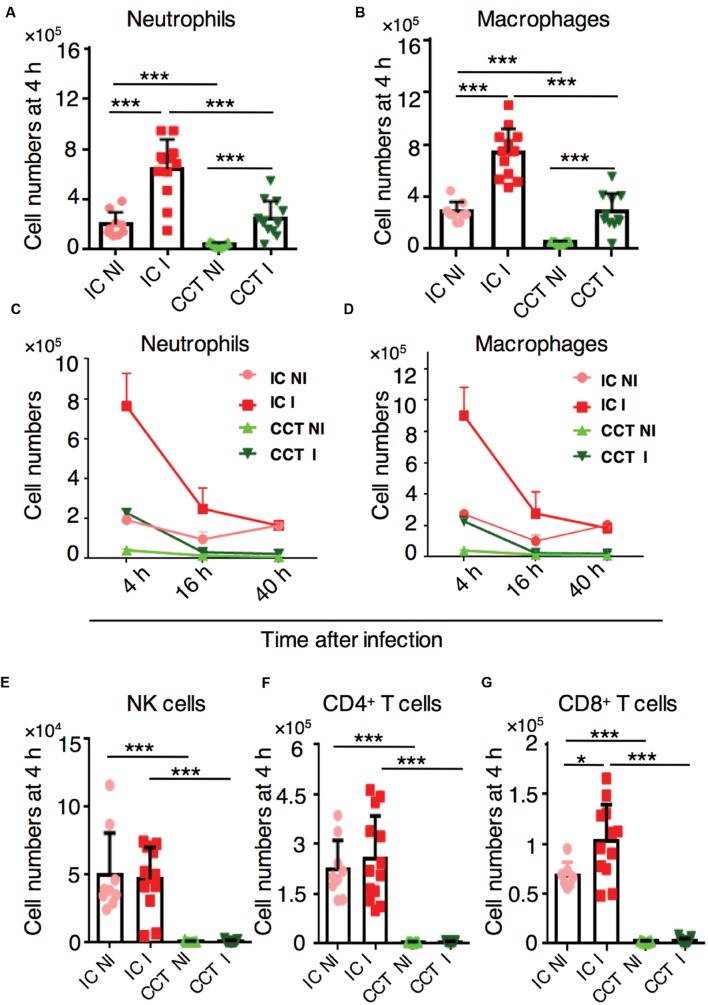
**Immune cell response in cyclophosphamide and cortisone treated (CCT) mice after *A. fumigatus* infection.** Flow cytometry of lungs from non-infected (NI) or infected (I) with 1 × 10^6^ *A. fumigatus* conidia immunocompetent (IC) and CCT mice at indicated time points, **(A)** *In vivo* lung neutrophil and (**B**) macrophage recruitment 4 h after *A. fumigatus* infection. **(C)** *In vivo* lung neutrophil recruitment 4, 16, and 40 h after *A. fumigatus* infection. **(D)** *In vivo* lung macrophage recruitment 4, 16, and 40 h after *A. fumigatus* infection. **(E)** *In vivo* lung NK cell, **(F)** CD4^+^ T cell **(G)** CD8^+^ T cell recruitment 4 h after *A. fumigatus* infection. Data are pooled from three independent experiments with at least *n* = 3/group of mice in each experiment. Unpaired Mann–Whitney *u*-test was utilized to determine significant differences: ^∗^*P* < 0.05; ^∗∗^*P* < 0.01; ^∗∗∗^*P* < 0.001.

To determine the timing and magnitude of immune cell recruitment at different stages of *A. fumigatus* infection in immunocompromised CCT mice, we infected them with 1 × 10^6^ *A. fumigatus* conidia intranasally and analyzed defined immune cell populations in the lungs at 4, 16, and 40 h p.i. by flow cytometry (**Supplementary Figure S1**). As determined previously, at these selected time points the fungus had evolved through different morphotypes (conidia, germlings, and hyphae, respectively) that would likely trigger distinct types of immune responses. All immune populations were strongly reduced in lungs of CCT mice when compared to the immune cells in lungs of immunocompetent mice at steady-state-conditions (**Figure 2**). Upon infection, myeloid cells, especially neutrophils (**Figure 2A**) and macrophages (**Figure 2B**) were significantly recruited to the lungs of CCT mice at 4 h p.i. However, cell numbers did not surmount numbers of non-infected immunocompetent mice under steady-state-conditions, suggesting that there were not sufficient cells to fight against infection. Despite their low number, these cells were strongly recruited at the 4 h p.i. time point; but not at 16 and 40 h p.i. (**Figures 2C,D**). We did not observe recruitment of other myeloid cells particularly monocytes, dendritic cells and eosinophils in CCT mice upon *A. fumigatus* infection (**Supplementary Figure S2**). Lymphoid cells, particularly NK cells, CD4^+^ T cells and CD8^+^ T cells were strongly reduced in the lungs of CCT mice and were not recruited upon *A. fumigatus* infection (**Figure 2E–G**), suggesting that lymphoid populations cannot play a pivotal role in the defense against *A. fumigatus* under these immunosuppressive conditions. To investigate underlying factors behind minute immune cell infiltration in CCT mice particularly at 16 and 40 h p.i., we performed TUNEL staining to observe apoptotic cells (**Supplementary Figure S3**). TUNEL positive cells appeared in CCT mice at 16 and 40 h p.i., whereas in IC mice no TUNEL positive cells were observed at 40 h p.i. Despite low or reduced recruitment at 16 and 40 h after infection, fungus growth was not controlled in CCT infected mice. Growing hyphae in the lung tissue might lead to apoptosis of some of the cells in CCT mice even at 40 h after infection, whereas in IC infected mice until 40 h fungus might have been cleared and no apoptotic cells were found in TUNEL staining.

### Myeloid Cells are Strongly Recruited to the Infected Lungs in CT Mice

**FIGURE 3:**
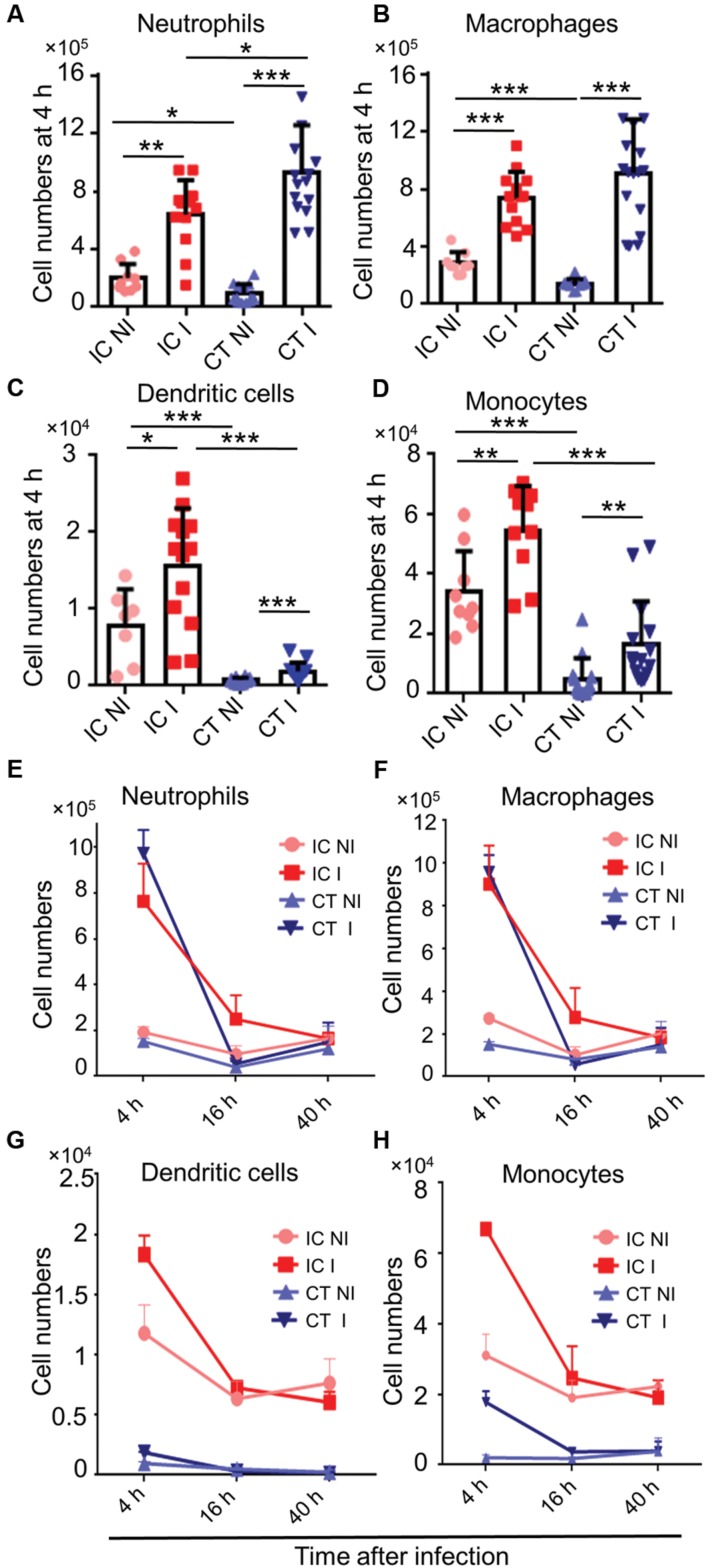
**Host immune cell response in corticosteroid treated (CT) mice after *A. fumigatus* infection.** Flow cytometric analysis of lungs from immunocompetent (IC) and CT mice non-infected (NI) or infected (I) with 1 × 10^6^ *A. fumigatus* conidia were euthanized at indicated time points. **(A)** *In vivo* neutrophil, (**B**) macrophage, **(C)** dendritic cell, and **(D)** monocyte recruitment to the lung at 4 h p.i. **(E–H)** *In vivo* recruitment of immune cells to the lung at 4, 16, and 40 h. p.i.: **(E)** neutrophils, **(F)** macrophages, **(G)** dendritic cells, and **(H)** monocytes. Data are pooled from three independent experiments with at least *n* = 3/group of mice in each experiment. Unpaired Mann–Whitney *u*-test was utilized to determine significant differences: ^∗^*P* < 0.05; ^∗∗^*P* < 0.01; ^∗∗∗^*P* < 0.001.

Corticosteroids are widely used immunomodulatory drugs in patients for a variety of clinical conditions (Shaikh et al., 2012). Corticosteroid treated mouse models are also employed to determine virulence of *A. fumigatus* mutants (Grahl et al., 2011). The phagocyte recruitment in corticosteroid treated mice after *A. fumigatus* infection had been previously studied (Duong et al., 1998; Balloy et al., 2005); however, the temporal kinetics of this dynamic immune cell response after *A. fumigatus* infection remains poorly defined. To determine the local host immune responses against *A. fumigatus* infection in CT mice, we infected CT mice with *A. fumigatus* conidia and analyzed immune cell recruitment at the above-specified time points of fungal development. Myeloid cells, particularly neutrophils (**Figure 3A**), macrophages (**Figure 3B**), dendritic cells (**Figure 3C**) and monocytes (**Figure 3D**) were recruited to the lungs of CT infected mice at 4 h p.i. Myeloid cell recruitment to lungs of infected mice was high at 4 h p.i. and low at 16 and 40 h p.i. (**Figures 3E–H**). Lymphoid cells were significantly reduced under these conditions and not recruited upon *A. fumigatus* infection (**Supplementary Figure S4**).

### Myeloid Cell Recruitment to Infected Lungs in CT Mice Correlates with Increase in Inflammatory Lung Cytokine Levels

**FIGURE 4:**
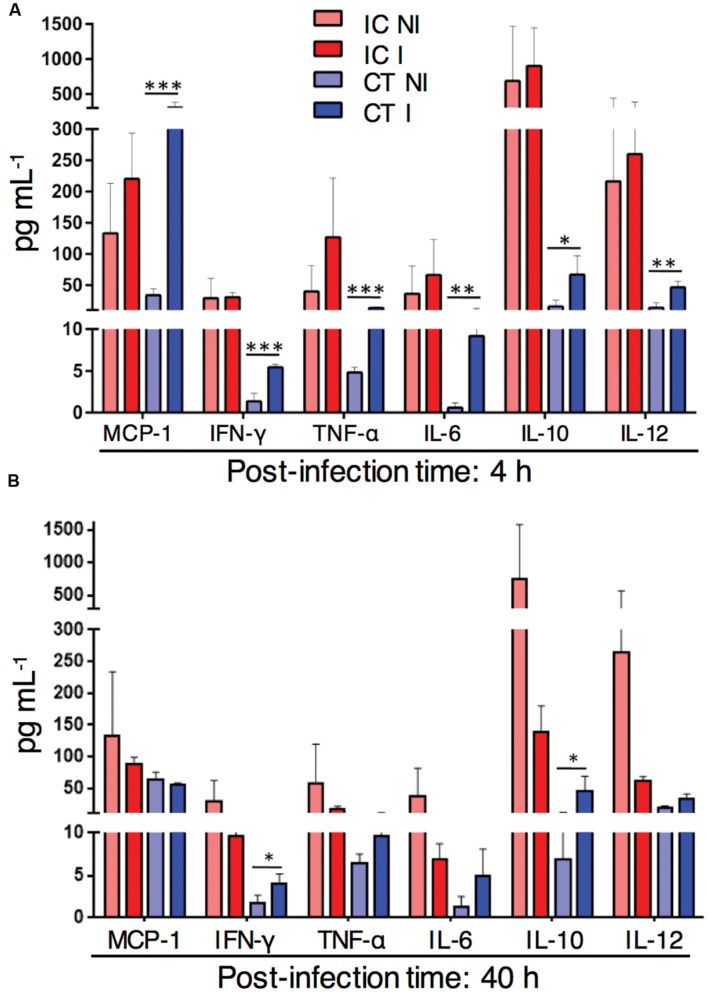
**Inflammatory cytokine response in corticosteroid treated (CT) mice after challenge with *A. fumigatus* conidia.** Cytometric Bead Array of lung homogenates from non-infected (NI) or with 1 × 10^6^ *A. fumigatus* conidia infected (I) immunocompetent (IC) and CT mice. **(A)** *In vivo* lung cytokine environment at 4 h after *A. fumigatus* infection. **(B)** *In vivo* lung cytokine environment at 40 h p.i. Data are representative of two independent experiments with *n* = 3 mice/group in each experiment. Unpaired Mann–Whitney *u*-test was utilized to determine significant differences: ^∗^*P* < 0.05; ^∗∗^*P* < 0.01; ^∗∗∗^*P* < 0.001.

Myeloid cells were strongly recruited to the infected lungs in corticosteroid treated mice. To determine the lung cytokine environment at different time points after *A. fumigatus* infection in corticosteroid treated mice, we measured inflammatory cytokines in lung homogenates of immunocompetent, CT infected and non-infected mice. At 4 h p.i. the amount of the inflammatory cytokines MCP-1, IFN-γ, TNF-α, IL-6, and IL-12 in CT infected mice significantly exceeded cytokine levels in CT non-infected mice (**Figure 4A**). At 40 h p.i. the levels of lung inflammatory cytokines, except IFN-γ were similar in both CT infected and non-infected mice. However, the amount of the anti-inflammatory cytokine IL-10 was significantly higher in CT infected mice compared to non-infected mice at both 4 and 40 h after infection (**Figure 4B**). In contrast to CT mice, inflammatory or anti-inflammatory cytokines were below detection limits to determine in lungs of CCT mice with or without infection by the multiplex assay. Strikingly, these results suggest that increased inflammatory response in CT mice after infection is accompanied by high lung myeloid cell recruitment to the CT infected lungs.

### Adoptively Transferred CD11b^+^ Myeloid Cells Rescue Cyclophosphamide Immunosuppressed Mice from Lethal *A. fumigatus* Infection

**FIGURE 5:**
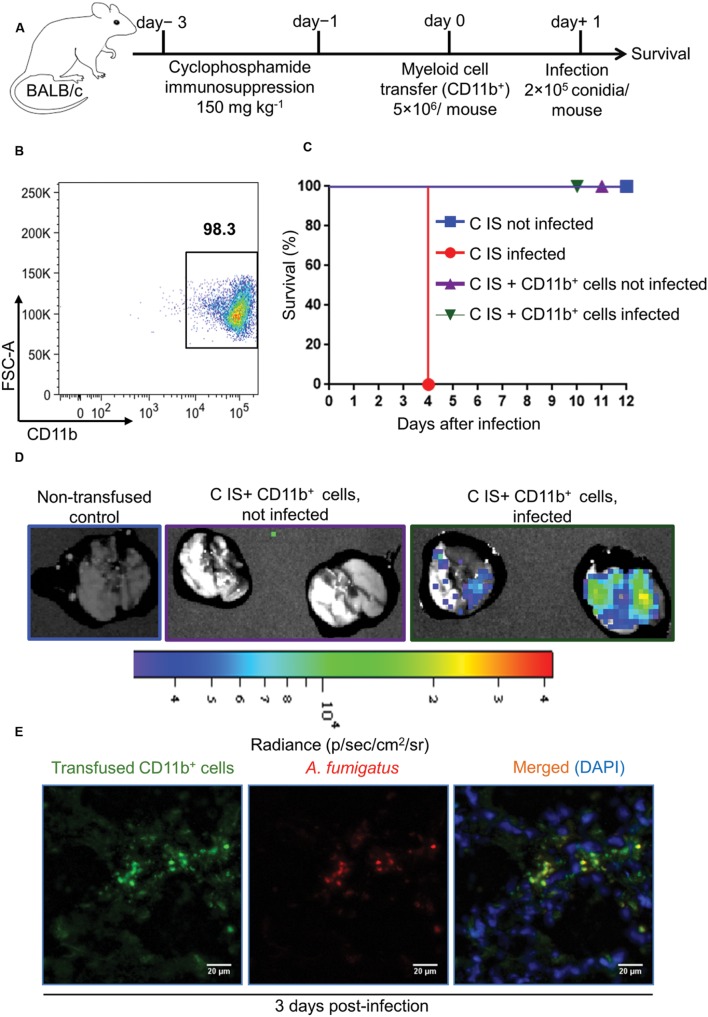
**Adoptive CD11b^+^ myeloid cell transfer protects cyclophosphamide immunosuppressed mice from lethal *A. fumigatus* infection.** **(A)** Experimental setup for adoptive CD11b^+^ myeloid cell transfer and *A. fumigatus* infection. Mice were immunosuppressed with cyclophosphamide on day -3 and day -1. On day 0, cyclophosphamide immunosuppressed (C IS) mice were injected with 5 × 10^6^ cells CD11b^+^ myeloid cells/mouse i.v. Subsequently, mice were intranasally infected with a lethal dose of *A. fumigatus* conidia (2 × 10^5^ conidia/mouse). **(B)** Purity of CD11b^+^ myeloid cells measured with flow cytometry after enrichment from bone marrow of tibia and femur bones. Cell purity always exceeded 95%. **(C)** Survival of C IS mice after *A. fumigatus* infection. C IS mice that had been transfused with CD11b^+^ myeloid cells completely resist an otherwise lethal *A. fumigatus* infection (*P* = 0.0003). All groups *n* = 8. Data are representative of three independent experiments *n* = 8/group of mice in each experiment. Log-rank (Mantel–Cox) test was utilized to determine survival significance. **(D)** Bioluminescence imaging. CD11b^+^ myeloid cells were enriched from L2G85 luciferase reporter mice and transfused to C IS mice and infected with TdTomato expressing *A. fumigatus*. *Ex vivo* bioluminescence imaging was performed 3 days p.i. *n* = 2 mice/ group. **(E)** Immunofluorescence microscopy at 3 days p.i. of lungs from C IS mice after transfused with luciferase expressing CD11b^+^ myeloid cells and infected with Afu-TdTomato conidia. *A. fumigatus* in red, anti-luciferase staining of transfused CD11b^+^ cells in green and DAPI staining for nuclei in blue color. Scale bar 20 μM.

**FIGURE 6:**
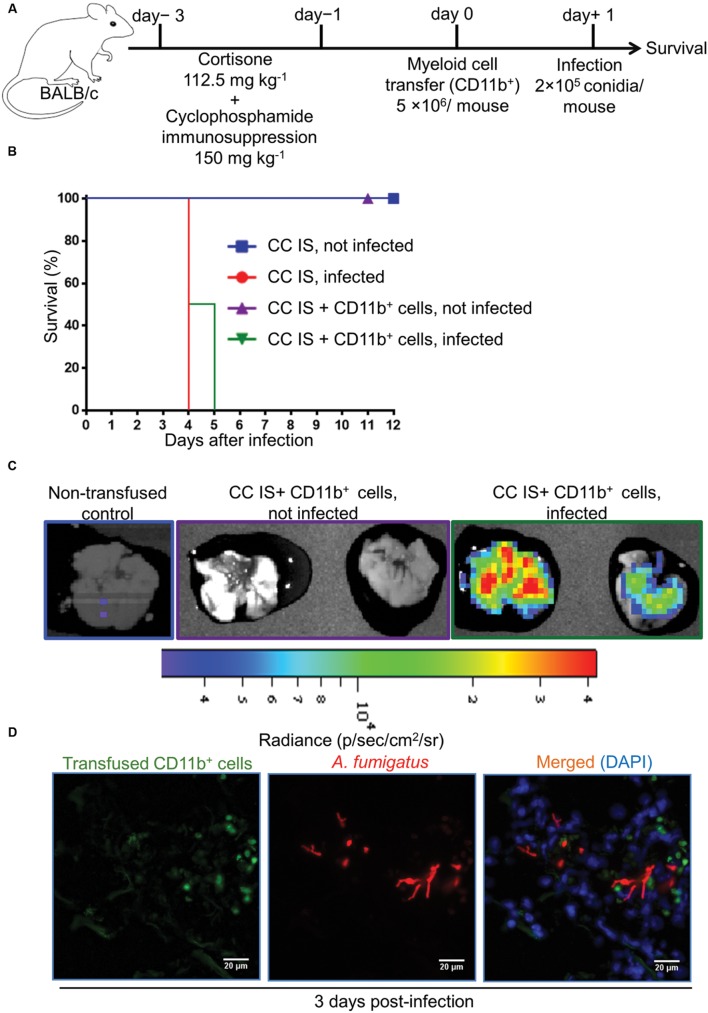
**Adoptively transferred CD11b^+^ myeloid cells do not protect from *A. fumigatus* infection if mice are immunosuppressed with both, cyclophosphamide and corticosteroids.** **(A)** Experimental setup for adoptive CD11b^+^ myeloid cell transfer and *A. fumigatus* infection. Mice were immunosuppressed with both, cyclophosphamide & cortisone on day -3 and day -1 (CC IS mice). On day 0, CC IS mice received 5 × 10^6^ CD11b^+^ myeloid cells i.v. and were intranasally infected with a lethal dose of 2 × 10^5^ *A. fumigatus* conidia to determine survival. **(B)** Survival of mice after adoptive CD11b^+^ myeloid cell transfer. Adoptive CD11b^+^ myeloid cell transfer does not protect CC IS mice from lethal *A. fumigatus* infection. No differences deemed significant [Log-rank (Mantel–Cox test)] between infected CC IS mice, and infected CC IS mice that had been transfused with CD11b^+^ myeloid cells. Data are representative of two independent experiment with *n* = 8/group of mice in each experiment. **(C)** Bioluminescence imaging. CD11b^+^ myeloid cells were enriched from L2G85 luciferase reporter mice and transfused into CC IS mice and infected with TdTomato expressing *A. fumigatus*. *Ex vivo* bioluminescence imaging was performed 3 days p.i. *n* = 2 mice/group. **(D)** Immunofluorescence microscopy of lungs from CC IS mice that had received luciferase expressing CD11b^+^ myeloid cells and were infected with Afu-TdTomato conidia at 3 days p.i. *A. fumigatus* in red, anti-luciferase staining for transfused CD11b^+^ cells in green and DAPI staining for nuclei in blue color. Scale bar 20 μM.

Regardless of the immune status of mice, myeloid but not lymphoid cells were recruited to the site of infection. Despite their strongly reduced immune cell numbers, this was also true for the lungs of CCT mice after *A. fumigatus* infection. To determine whether myeloid cells alone can rescue immunosuppressed mice from lethal *A. fumigatus* infection we adoptively transferred CD11b^+^ myeloid cells into immunosuppressed mice that had been treated with cyclophosphamide (150 mg/kg) on days -3 and -1 (**Figure 5A**) alone, since corticosteroid models might interfere with antifungal functions of myeloid cells, as CT infected mice were not resistant to infection irrespective to strong myeloid cell recruitment to the lungs. CD11b^+^ myeloid cells were enriched from bone marrow of BALB/c donor mice (**Figure 5B**) and transfused intravenously to cyclophosphamide immunosuppressed (C IS) mice on day 0. This CD11b^+^ population consisted of CD11b^+^Ly6G^high^ neutrophils (70 ± 1%), CD11b^+^Ly6G^dim^ cells (5 ± 0.5%), CD11b^+^Ly6G^-^Ly6C^+^ monocytes (7 ± 1%) and CD11b^+^Ly6G^-^Ly6C^-^ non-differentiated neutrophilic and monocytic precursor cells (18 ± 4%, **Supplementary Figure S5**). On day +1 we infected mice with a lethal dose of 2 × 10^5^ *A. fumigatus* conidia and monitored their survival (**Figure 5C**). C IS mice, which had received an adoptive CD11b^+^ myeloid cell transfer, were resistant to a lethal infection dose, whereas, immunosuppressed and infected (control) mice were unable to clear the infection and died within 4 days after infection (**Figure 5C**). To determine whether transfused CD11b^+^ myeloid cells recruit to the infected lungs and directly impair *A. fumigatus* growth, we performed an adoptive cellular transfer experiment with transgenic firefly luciferase expressing CD11b+ myeloid cells enriched from a BALB/c.L2G85 luciferase reporter mouse (Cao et al., 2004; Beilhack et al., 2005) and infected with TdTomato expressing *A. fumigatus* conidia with the same experimental settings as described in **Figure 5**. The transfused CD11b^+^ cells were detected in C IS infected and not infected lungs 3 days p.i. with ex vivo bioluminescence imaging (Chopra et al., 2015). Lungs from transfused and infected C IS mice contained many CD11b^+^ cells, whereas lungs from transfused and not infected C IS mice did not show CD11b^+^ myeloid cells (**Figure 5D**). To determine whether recruited CD11b^+^ myeloid cells interacted with *A. fumigatus*, we performed fluorescence microscopy on C IS transfused and infected lung sections. Luciferase expressing CD11b^+^ cells were found in close proximity to *A. fumigatus* and fungal hyphal formation was impaired at 3 days p.i. (**Figure 5E**). These results indicate that adoptively transferred CD11b^+^ cells recruit to the infected lungs and support locally the control of *A. fumigatus* fungal growth. In contrast, cortisone and cyclophosphamide immunosuppressed (CC IS) mice, which had received adoptively transferred CD11b^+^ myeloid cells, could not clear the infection and died within 5 days after infection (**Figures 6A,B**). CC IS mice which had received luciferase expressing CD11b^+^ myeloid cells showed strong influx of these cells to the lungs upon infection (**Figure 6C**). However, these strongly recruited cells failed to control of *A. fumigatus* growth in CC IS infected lungs (**Figure 6D**). These striking results indicate that corticosteroid treatment might either have caused tissue damage to recipient mice or affected the protective function of adoptively transferred CD11b^+^ myeloid cells. However, myeloid cells significantly contributed to the host anti-*A. fumigatus* defense as adoptive transfer of CD11b^+^ myeloid cells alone rescued cyclophosphamide immunosuppressed mice from lethal *A. fumigatus* infection.

## Discussion

The pivotal role of the innate immune system eliminating *A. fumigatus* conidia in healthy individuals has long been well-recognized (Balloy and Chignard, 2009). The anatomical and physiological barriers of the respiratory tract restrict most of the airborne conidia to reach alveolar spaces, however, the small size and hydrophobic nature of conidia, strongly favors some of them to cross alveolar epithelia and reach alveolar spaces (Margalit and Kavanagh, 2015). Most of the conidia in alveolar spaces are eradicated by resident phagocytes without any further development of antibody-or-cell mediated acquired immunity (Park and Mehrad, 2009). However, a compromised immune system provide the basis for germination of *A. fumigatus* conidia and subsequent lung infections (Margalit and Kavanagh, 2015). In the last few decades, several studies defined the anti-*A. fumigatus* functions of innate or adaptive immune cells (Cramer et al., 2011; Sales-Campos et al., 2013). Most of the *in vivo* studies focused on depleting a defined immune cell population from healthy murine models to determine the consequences of loss of distinct cell populations on survival and overall outcome of the disease. Nevertheless, to improve management and treatment of *A. fumigatus* lung infections in immunocompromised patients it is essential to study host pathogen interactions in murine models of aspergillosis that mimic scenarios of immunocompromised patients. Moreover, corticosteroid treated (CT) and corticosteroid and cyclophosphamide treated (CCT) mice are often used for virulence analysis of *A. fumigatus* mutants. CT or CCT models are selected for virulence analysis depending on the observed phenotype of the fungal mutant, for instance CT models are often used for virulence analysis of auxotroph mutants and CCT models for oxidative stress mutants (Sheppard et al., 2005; Chiang et al., 2008; Spikes et al., 2008; Amich et al., 2013; Staats et al., 2013). However, the immune status of these models under steady-state and infected conditions remained largely elusive.

Here, we employed these two clinically relevant immunocompromised murine models to study the host immune responses after *A. fumigatus* challenge. In the CCT model, mice received the combination of cyclophosphamide and corticosteroid treatment. This combination is widely used in treating patients with idiopathic pulmonary fibrosis (Collard et al., 2004; Kawasumi et al., 2015), acute/subacute interstitial pneumonia (Kameda et al., 2005), refractory optic neuritis in Wegener’s granulomatosis (Huchzermeyer et al., 2013) and light chain (AL) amyloidosis (Palladini et al., 2015). However, treatment with this combination is widespread across several clinical situations; the risk of *A. fumigatus* infections associated with this treatment and immune cell responses after *A. fumigatus* infection during the treatment remained poorly defined. We confirmed that CCT mice were highly susceptible to *A. fumigatus* infection and died from infection within 4 days after *A. fumigatus* challenge. Severe leukopenia permits rapid colonization of *A. fumigatus* characterized by elongated hyphae in lung tissue 40 h after infection resulting in death of CCT infected mice within 4 days after infection, which is consistent with previous findings (Amich et al., 2013). Nevertheless, despite their strongly reduced number, myeloid cells, particularly neutrophils and macrophages were recruited to the infected lungs in CCT mice. Myeloid cell numbers in the lungs of CCT infected mice did not exceed numbers found in lungs of immunocompetent mice under steady-state conditions, which indicates that there were not sufficient myeloid cells recruited to the infected lungs in CCT mice to clear the infection or prolong the life span of CCT mice. Inflammatory cytokine responses are crucial for properly resolving an *A. fumigatus* lung infection (Chotirmall et al., 2013), for instance, TNFα initially released from alveolar macrophages and later by recruited neutrophils and monocytes is important to clear *A. fumigatus* infection (Mehrad et al., 1999a,b; Brieland et al., 2001; Palladino et al., 2003). Other proinflammatory cytokines, such as IL-6, MCP-1, and IFNγ have been described as vital to eliminate pulmonary *A. fumigatus* infections (Blease et al., 2001; Brieland et al., 2001; Cenci et al., 2001). All the above-mentioned cytokines were undetectable in CCT mice, both, under steady-state conditions as well as after *A. fumigatus* infection. These results support the strong immunosuppressive action of high-doses of cyclophosphamide causing the high susceptibility of CCT mice to lethal *A. fumigatus* infection. The combination of cyclophosphamide and corticosteroid treatment strongly reduced lymphoid cells in CCT mice and no lymphoid cells were recruited upon infection. Infection related risk is very high with this type of treatment and clinicians might need to take special precautions to avoid infections by *A. fumigatus* throughout the treatment period.

Corticosteroids are widely prescribed drugs in several clinical situations (Barnes, 2006; Emadi et al., 2009). We showed that corticosteroid treated mice survived for 7 days after *A. fumigatus* challenge, which correlates with greater myeloid cell recruitment and inflammatory lung cytokines such as MCP-1, IFN-γ, TNFα and IL-6 levels in the infected lungs. However, survival after infection was not greatly improved when compared to CCT infected mice suggesting that corticosteroids may rather affect anti-fungal functions of immune cells than influencing direct myeloid cell recruitment. Clearly, further studies are warranted to completely understand the effects of corticosteroids on myeloid cells on anti-*A. fumigatus* defense functions. Our results are also in line with the observation that corticosteroid treatment causes strong inflammation, which might enhance tissue damage after infection (Ibrahim-Granet et al., 2010; Grahl et al., 2011) leading to death within 7 days after infection. Overall, in both, CCT and CT models myeloid but not lymphoid cell infiltration of the lungs dominated after *A. fumigatus* infection.

Importantly, CD11b^+^ myeloid cells alone rescued cyclophosphamide immunosuppressed mice from lethal *A. fumigatus* infection. The adoptive transfer of common myeloid progenitors (CMP) and granulocyte-monocyte progenitors (GMP) protected mice against disseminated *A. fumigatus* infections (BitMansour et al., 2002). This protection was only conferred when mice were infected on day +7 (67% survival) or day +11 (100% survival) after transplantation. None of the mice survived when infected on day +3 after transplantation (BitMansour et al., 2002). The adoptive transfer of common myeloid progenitor cells bears the benefit to provide immune-reconstitution for longer time periods, yet their requirement to firstly home to the bone marrow for further development into mature effector cells delays the host defense against *A. fumigatus* infection. The adoptive transfer of bulk CD11b^+^ myeloid cells bears the advantage that it is technically simple to achieve through enrichment with magnetic beads and it proved effective to provide early protection from an otherwise lethal *A. fumigatus* infection. The surface receptor CD11b (integrin alpha M, ITGAM) subunit forms the heterodimeric integrin αMβ2 integrin, which is expressed on a variety of myeloid cells including neutrophils, monocytes, and macrophages. These immune populations play a pivotal role in defense against lethal *A. fumigatus* lung infections (Balloy and Chignard, 2009). For instance, the myeloid subset of neutrophils has been shown to be critical in controlling *A. fumigatus* infection (Feldmesser, 2006). The timing of neutrophil recruitment is vital for *A. fumigatus* clearance as a small delay in neutrophils arrival leads to increased disease susceptibility (Mehrad et al., 1999a; Bonnett et al., 2006). Macrophages are effective phagocytic cells and important for fungal pathogen clearance (Bhatia et al., 2011). Finally, circulating monocytes are major precursor cells, once they become activated with the infectious stimulus they develop into macrophages and dendritic cells (monocyte derived dendritic cells) and play an important role in elimination of *A. fumigatus* infections (Espinosa et al., 2014). The CD11b^+^ myeloid cells were transfused into immunosuppressed mice and then infected with *A. fumigatus*. However, particularly for the purpose of clinical translation, it might be interesting to perform future adoptive transfer experiments in mouse models with already established invasive aspergillosis. In clinical scenarios, cellular immunotherapy still remains an intriguing therapeutic option for patients suffering from invasive *A. fumigatus* infections who do not respond to conventional antifungal drugs. Our adoptive CD11b^+^ myeloid cell transfer experiments provide a basis for the future development of novel myeloid based immunotherapy. Clearly, further experiments are required to establish the optimal dosage and timing for CD11b^+^ myeloid cell transfusion to treat already established invasive infections and to address transfusion related side effects. In contrast to effective infection control after adoptive CD11b^+^ myeloid cell transfer into otherwise highly susceptible cyclophosphamide treated mice, adoptive CD11b^+^ myeloid cell transfer did not protect corticosteroid and cyclophosphamide immunosuppressed mice from lethal infection. This clearly suggests that corticosteroid treatment enhances inflammation mediated tissue damage or impairs antifungal functions of myeloid cells. Further studies are warranted to dissect these mechanisms and to address the effects of corticosteroids on antifungal functions of immune cell subsets.

Collectively, we provided a comprehensive analysis of immune cell responses after *A. fumigatus* infection in two clinically relevant immunocompromised mouse models. These models of invasive aspergillosis along with detailed information of immune cell response after *A. fumigatus* infection might also help in testing the efficacy of non-conventional novel anti-fungal therapies to treat invasive *A. fumigatus* infections, for instance new small molecule inhibitors, antibodies or therapeutic RNAs (Kalleda et al., 2013). Since we showed successful control of *A. fumigatus* infection after adoptive transfer of CD11b^+^ myeloid cells into cyclophosphamide immunosuppressed mice, our results confirm that CD11b^+^ myeloid cells are major contributors to fight against *A. fumigatus* lung infections in immunocompromised conditions. These results may further support the future development of novel myeloid-based immunotherapies against opportunistic fungal infections.

## Author Contributions

NK, JA, and AB designed the study. NK, JA, BA, and KM carried out experiments. NK, JA, HE, SP, MB, KH, ZM, and AB analyzed the data. NK wrote the manuscript. NK, JA, HE, SP, MB, KH, ZM, and AB revised the manuscript and all the authors approved the final manuscript.

## Conflict of Interest Statement

The authors declare that the research was conducted in the absence of any commercial or financial relationships that could be construed as a potential conflict of interest.
