# Supplementary material for: Dynamic Immune Cell Recruitment After Murine Pulmonary Aspergillus fumigatus Infection under Different Immunosuppressive Regimens
Source: Front Microbiol. 2016 Jul 13;7:1107. doi: 10.3389/fmicb.2016.01107 (PMC4942482; doi:10.3389/fmicb.2016.01107)
Supplement: Supplementary file 1 [file Image_1.PDF]

Supporting Information:
Dynamic immune cell recruitment after murine pulmonary *Aspergillus fumigatus* infection under different immunosuppressive regimens

Authors: Natarajaswamy Kalleda[1,2,3,4], Jorge Amich[1,2], Berkan Arslan[1], Spoorthi Poreddy[5], Katharina Mattenheimer[1], Zeinab Mokhtari[1], Hermann Einsele[1], Matthias Brock[6,7], Katrin Heinze[8] and Andreas Beilhack[1,2,3,4]*

Affiliations:

[1]Department of Medicine II, Würzburg University Hospital, Würzburg, Germany.

[2]Research Center for Infectious Diseases, Julius-Maximilians-University Würzburg, Würzburg, Germany.

[3]Interdisciplinary Center for Clinical Science (IZKF) Research Laboratory, Würzburg, Germany

[4]Graduate School of Life Sciences Würzburg, Germany

[5]Max Planck Institute for Chemical Ecology, Jena, Germany.

[6]Leibniz Institute for Natural Product Research and Infection Biology, Hans Knoell Institute; Institute for Microbiology, Friedrich Schiller University Jena, Germany.

[7]Fungal Genetics and Biology Group, University of Nottingham, School of Life Sciences, University Park, NG7 2RD, Nottingham, UK.

[8]Rudolf Virchow Center. Julius-Maximilians-University Würzburg, Würzburg, Germany.

Running title: *Aspergillus fumigatus* and immune response

Keywords: *Aspergillus fumigatus*, immune cell recruitment, $CD11b^+$ myeloid cells, corticosteroids and cyclophosphamide.

***Correspondence to:**

Andreas Beilhack

beilhack_a@ukw.de

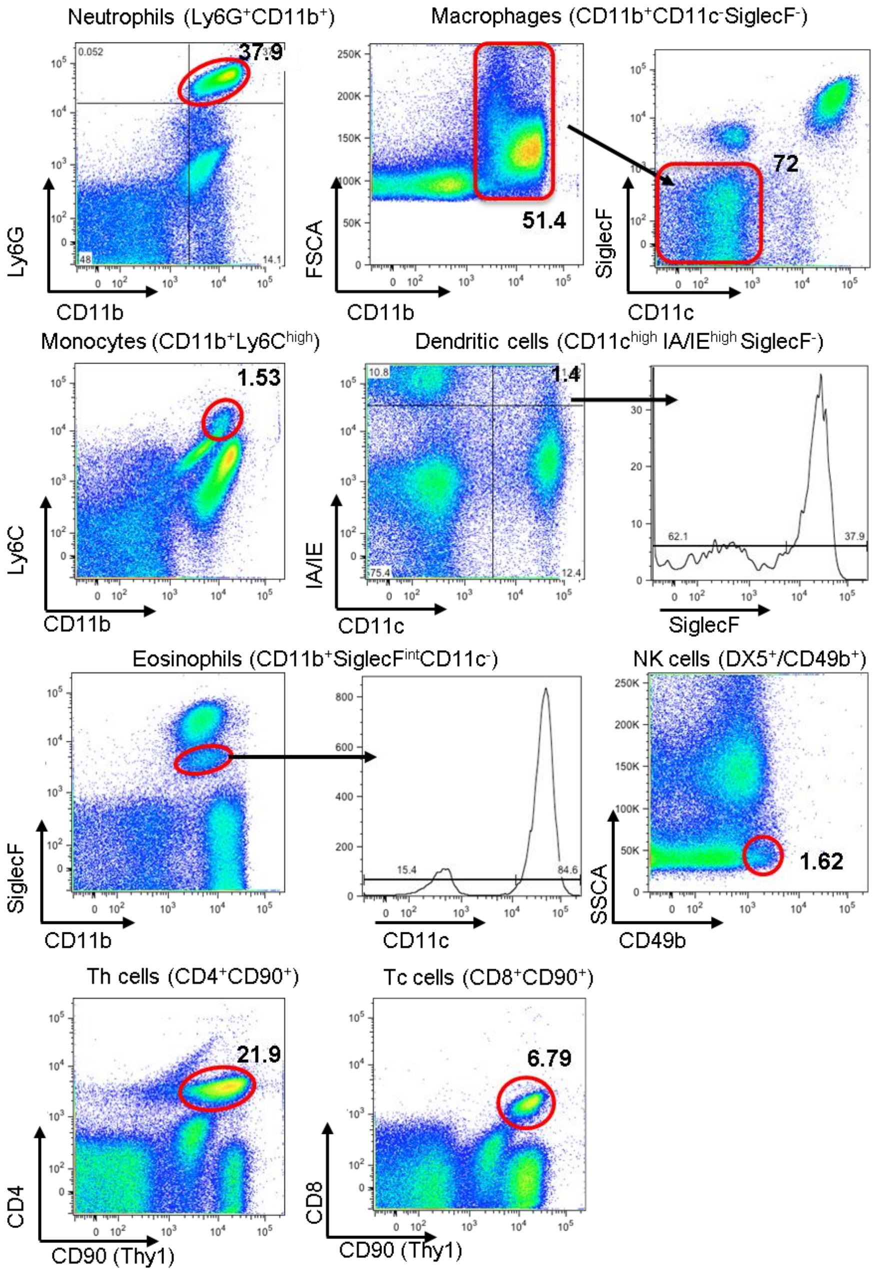

Figure. S1. Flow cytometry gating strategy for immune cell populations in the lung. Representative dot plots show distinct immune cell phenotypes based on defined antibody stainings.

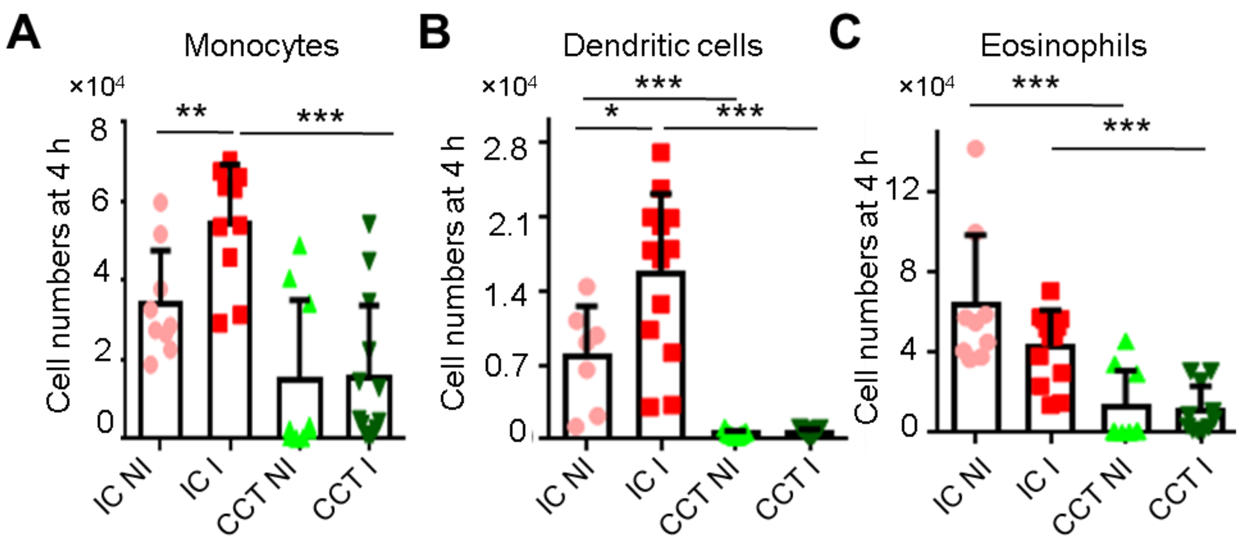

Fig. S2 Immune cell response in CCT mice after A. fumigatus infection. Flow cytometry of lungs from non-infected (NI) or with 106 A. fumigatus conidia infected (I) immunocompetent (IC) and Cyclophosphamide & corticosteroid treated (CCT) mice at indicated time points, (A) In vivo lung monocyte recruitment 4 h after A. fumigatus infection. (B) In vivo lung dendritic cell recruitment 4 h after A. fumigatus infection. (C) In vivo lung eosinophil recruitment 4 h after A. fumigatus infection. Data are pooled from three independent experiments with at least n=3/ group of mice in each experiment. Unpaired Mann-Whitney *u*-test was utilized to determine significant differences: * P<0.05; ** P<0.01; *** P<0.001.

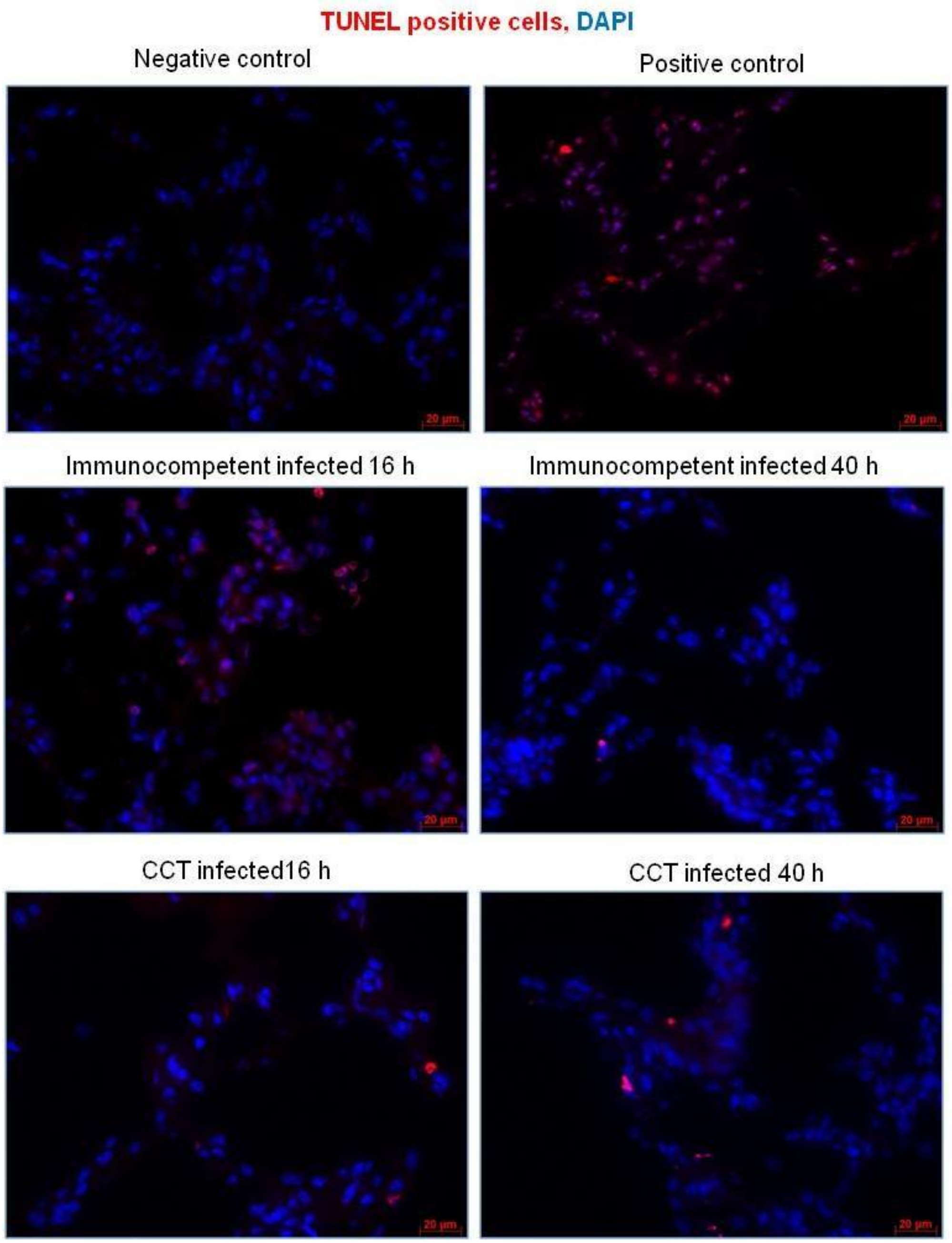

Fig. S3. Detection of apoptotic cells by TUNEL staining. Lung sections of immunocompetent (IC) and corticosteroid & cyclophosphamide treated (CCT) mice at 16 h and 40 h after infection were prepared and TUNEL staining was performed using a commercial kit (TUNEL positive cells in red, DAPI staining for nuclei in blue, Scale bar 20µM).

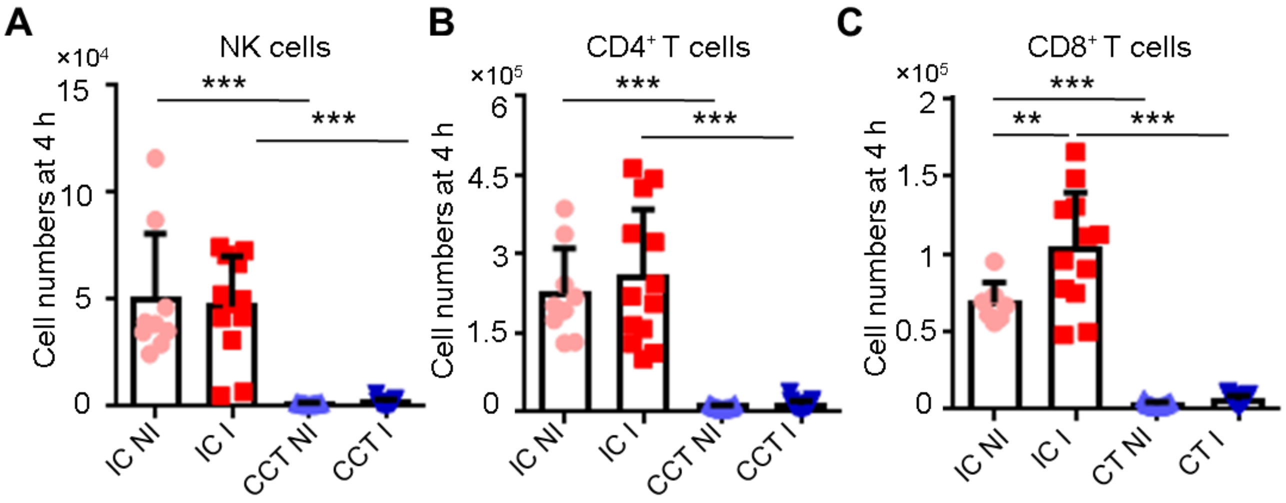

Fig. S4 Immune cell response in CT mice after A. fumigatus infection. Flow cytometry of lungs from immunocompetent (IC) and corticosteroid treated (CT) mice, either non-infected (NI) or 4 hours after infection (I) with 106 A. fumigatus conidia. (A) In vivo lung NK cell (B) In vivo lung CD4+ T cell and (C) In vivo lung CD8+ T cell recruitment 4 h after A. fumigatus infection. Data are pooled from three independent experiments with at least n=3/ group of mice in each experiment. Unpaired Mann-Whitney *u*-test was utilized to determine significant differences: * P<0.05; ** P<0.01; *** P<0.001.

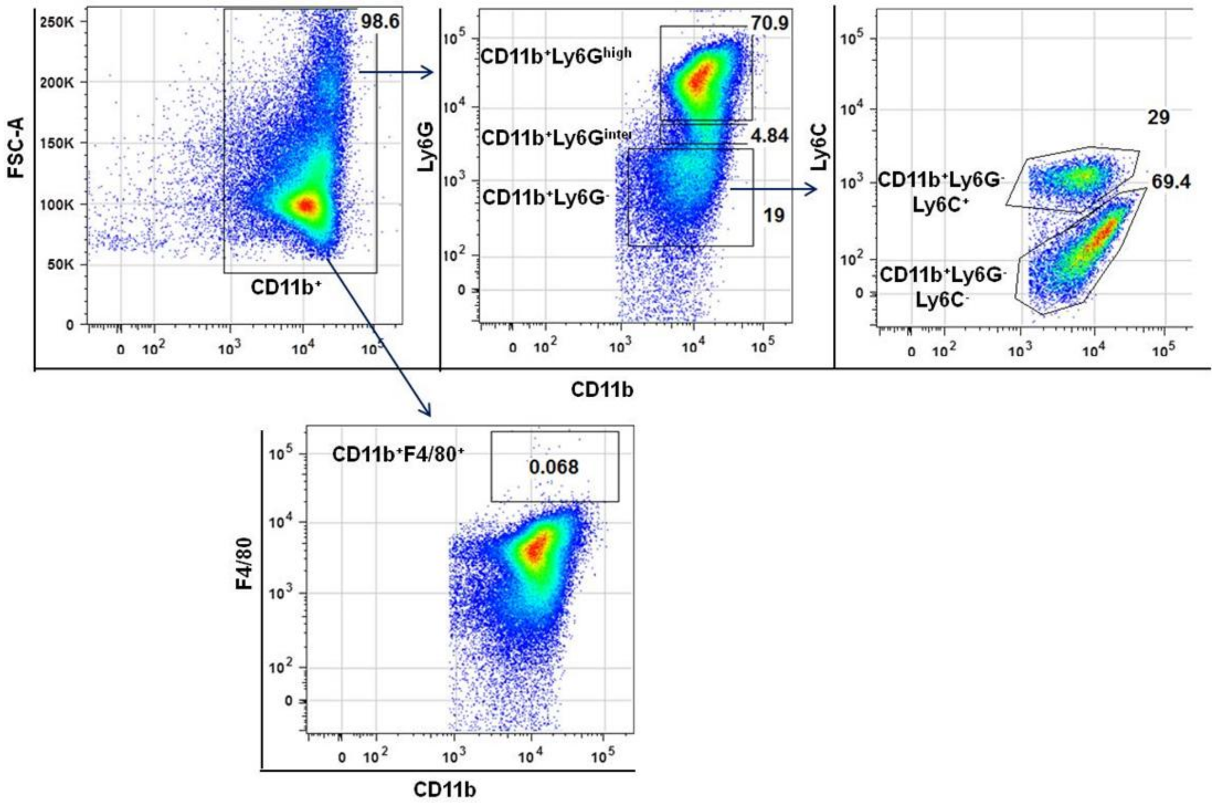

Fig.S5. Flow cytometry gating strategy for CD11b+ enriched myeloid cell fraction. CD11b+ cells were enriched from mouse bone marrow using a CD11b isolation kit. Dot plots show distinct immune cell phenotypes (CD11b+Ly6Chigh: monocytes, CD11b+Ly6Ghigh: neutrophils, CD11b+F4/80: macrophages) based on defined antibody stainings.
